# Foot Position Measurement during Assistive Motion for Sit-to-Stand Using a Single Inertial Sensor and Shoe-Type Force Sensors

**DOI:** 10.3390/ijerph181910481

**Published:** 2021-10-06

**Authors:** Kodai Kitagawa, Ibai Gorordo Fernandez, Takayuki Nagasaki, Sota Nakano, Mitsumasa Hida, Shogo Okamatsu, Chikamune Wada

**Affiliations:** 1 Graduate School of Life Science and Systems Engineering, Kyushu Institute of Technology, 2–4 Hibikino, Wakamatsu-ku, Kitakyushu 808-0196, Japan; fernandez.ibai-gorordo625@mail.kyutech.jp (I.G.F.); hidam@kawasakigakuen.ac.jp (M.H.); shogo.okamatsu182505@gmail.com (S.O.); wada@brain.kyutech.ac.jp (C.W.); 2 Department of Rehabilitation, Tohoku Bunka Gakuen University, 6-45-1 Kunimi, Aoba-ku, Sendai 981-8551, Japan; nagasaki@rehab.tbgu.ac.jp; 3 Department of Rehabilitation, Kyushu University of Nursing and Social Welfare, 888 Tomio, Tamana 865-0062, Japan; nakano@kyushu-ns.ac.jp; 4 Department of Physical Therapy, Osaka Kawasaki Rehabilitation University, 158 Mizuma, Kaizuka 597-0104, Japan; 5 Department of Physical Therapy, Kitakyushu Rehabilitation College, 1575 Kamikatashima, Kanda-machi, Miyako-gun 800-0343, Japan

**Keywords:** foot position, assistive motion, sit-to-stand, wearable sensor, force sensor, inertial sensor, machine learning technique

## Abstract

Assistive motion for sit-to-stand causes lower back pain (LBP) among caregivers. Considering previous studies that showed that foot position adjustment could reduce lumbar load during assistive motion for sit-to-stand, quantitative monitoring of and instructions on foot position could contribute toward reducing LBP among caregivers. The present study proposes and evaluates a new method for the quantitative measurement of foot position during assistive motion for sit-to-stand using a few wearable sensors that are not limited to the measurement area. The proposed method measures quantitative foot position (anteroposterior and mediolateral distance between both feet) through a machine learning technique using features obtained from only a single inertial sensor on the trunk and shoe-type force sensors. During the experiment, the accuracy of the proposed method was investigated by comparing the obtained values with those from an optical motion capture system. The results showed that the proposed method produced only minor errors (less than 6.5% of body height) when measuring foot position during assistive motion for sit-to-stand. Furthermore, Bland–Altman plots suggested no fixed errors between the proposed method and the optical motion capture system. These results suggest that the proposed method could be utilized for measuring foot position during assistive motion for sit-to-stand.

## 1. Introduction

### 1.1. Lower Back Pain due to Assistive Motion for Sit-to-Stand

Assistive motion for sit-to-stand causes lower back pain (LBP) among caregivers due to heavy manual lifting [1,2]. Smedley et al. reported that 69% of nurses experienced LBP caused by patient handling tasks, including assistive motion for sit-to-stand [1]. Furthermore, our investigation on occupational injury revealed that caregivers experience LBP caused by manual lifting for assistive motion [2]. Although stationary lifting devices can be used to prevent LBP, several devices cause upper limb discomfort during the sitting up process [3]. Moreover, these stationary devices cannot be used in all workspaces owing to several limitations such as cost, time, efficiency, and space. Thus, ergonomic interventions to reduce lumbar load without stationary devices such as posture adjustment are necessary to prevent LBP during manual lifting.

### 1.2. Related Works to Prevent LBP due to Assistive Motion for Sit-to-Stand

Manual lifting is considered to be an assistive motion for sit-to-stand. Posture adjustment based on ergonomics has been found to be effective for reducing lumbar load during manual lifting [4]. Trunk angle and foot position have been considered important posture parameters for reducing the lumbar load during manual lifting [5,6,7,8]. Hoogendoorn et al. reported that trunk flexion and rotation are risk factors for the development of LBP [5], whereas Delisle et al. found that strategic foot placement might improve lumbar load during manual handling [6,7]. Our previous studies suggest that the adjustment of the quantitative foot position could reduce the lumbar load during assistive motion for sit-to-stand [8,9]. A previous investigation using computational musculoskeletal simulation revealed that foot position with long anteroposterior foot distance can reduce the compression stress in the L4–L5 joint [9]. Furthermore, a previous study that used electromyography revealed that the optimal foot position (foot distances in the anteroposterior and lateromedial directions at 55% and 20% of the body height, respectively) provided the use of the lower limb instead of the lumbar spine during assistive motion for sit-to-stand. [8]. Foot position is considered an effective posture parameter because it can be easily adjusted in the initial posture [8,9].

The aforementioned investigations therefore suggest that trunk angle and foot position monitoring and guidance can contribute to the prevention of LBP during manual handling. Previous studies have developed monitoring systems for trunk angle during manual handling [10,11,12]. The PostureCoach can measure and provide feedback on trunk angle during manual handling using wearable inertial sensors [10,11]. Training programs using this system can improve the trunk angle of caregivers during manual care activities [11]. Moreover, Cerqueira et al. developed a smart vest for real-time feedback to prevent work-related musculoskeletal disorders [12]. This smart vest can measure and provide feedback regarding trunk, neck, and arm postures using inertial sensors and haptic motors [12]. As mentioned earlier, several trunk angle monitoring systems using wearable sensors are available. On the other hand, a previous study showed that posture adjustment to prevent LBP requires both a real-time feedback system for trunk angle and verbal instruction from a personal trainer that is related to lower limb use such as “use legs instead of back” [13]. This study indicates that lower limb status should be also monitored as well as trunk angle to realize posture adjustment using a wearable system alone. However, no wearable monitoring system has focused on foot position during manual lifting.

### 1.3. Related Studies for Foot Position Measurement

Generally, foot position is measured using vision-based systems, such as optical motion capture systems and video cameras [14,15,16], which cannot be used in actual workspaces due to the large spaces occupied by these systems and the limited measurement areas that are available. Therefore, the measurement of foot position for the prevention of LBP during assistive motion for sit-to-stand must be achieved using a small number of wearable sensors. During gait analysis, the foot trajectory is often measured using an inertial sensor on the shoe [17]. However, integral errors have been a concern when directly calculating long-term foot trajectories using inertial data obtained from the inertial sensor [17]. To reduce integral errors of the inertial sensor, more than 10 algorithms have been developed [17]. However, these algorithms cannot be applied to assistive motion for sit-to-stand given that they are based on specific and frequent gait events [17]. Although an ultrasonic sensor can also be used to measure foot position, these sensors cannot measure foot position when obstacles are present between both feet [18].

### 1.4. Potential of Inertial Sensor and Shoe-Type Force Sensors

To address the aforementioned concerns, we have been developing a method for measuring foot position using a single inertial sensor on trunk and shoe-type force sensors [19]. Previous studies have shown that the use of an inertial sensor on the trunk and shoe-type force sensors can be considered an effective method for measuring foot position given the relationship between trunk movement and foot position during manual handling [9,20,21,22,23]. Wagner et al. found that a wide-footed stance is related to trunk rotation during manual lifting tasks [20]. Kingma et al. reported that a wide-footed stance increased trunk bending [21]. Our previous study indicated that foot position with a long anteroposterior distance reduced trunk bending [9]. From these relationships, we hypothesized that foot position can be estimated using inertial data obtained from the inertial sensor on the trunk.

Furthermore, Jeong et al. suggested that the foot position during manual lifting affects the ground reaction forces on the toe and heel and the trajectory of the center of pressure (COP) [22]. Lee et al. revealed that the COP shifted to the left side when the right foot was placed forward [23]. Moreover, previous studies have shown that shoe-type force sensors could be used to recognize occupational postures, including different foot positions [24,25], and to monitor trunk movement, lumbar load, and arm movement during manual handling [19,25,26,27]. From these findings, we hypothesized that foot position can be estimated on the basis of the distribution of the ground reaction force on the foot that is obtained from shoe-type force sensors. Therefore, a single inertial sensor on the trunk and shoe-type force sensors were selected as suitable wearable sensors to estimate the foot position.

### 1.5. Potential of Machine Learning-Based Regression

A single inertial sensor on the trunk and shoe-type force sensors could not measure the quantitative foot position [19]. Thus, the current study utilized machine learning-based regression algorithms for the indirect wearable measurement of quantitative foot position. Machine learning-based regression algorithms have previously been applied for indirect measurements using a small number of wearable sensors [28]. Furthermore, machine learning can be applied to estimate nonlinear relationships [28]. Moreover, machine learning can be used in applications using wearable sensors that require indirect estimations for joint movement, muscle activity, and position [28]. Recent studies have shown that the combination of wearable sensors and machine learning could be used to estimate kinematic values during lifting and squatting [25,26,29]. Antwi-Afari et al. reported that several lifting postures could be identified using machine learning and that foot pressure distributions could be obtained from insole force sensors [25]. Matijevich et al. found that lumbar load during manual lifting could be predicted by a machine learning-based regression model with an inertial sensor and shoe-type force sensors [26]. Choffin et al. estimated foot angle while squatting using machine learning and insole force sensors [29]. These previous studies focused on tasks that are similar to our current research, and thereby provided insight into the potential usefulness of the combination of wearable sensors and machine learning for estimating foot position during manual lifting [25,26,29]. From these findings, we hypothesized that machine learning is the most suitable method for the indirect estimation of foot position.

### 1.6. Objective of This Paper

The present study therefore aimed to propose and evaluate a new method for measuring foot position during manual lifting using a machine learning technique and only a single inertial sensor on the trunk and shoe-type force sensors.

### 1.7. Contribution of This Paper

Previous wearable systems to prevent LBP could monitor and provide feedback for injury risks such as lumbar loads and trunk angles during manual lifting [10,11,12]; moreover, other previous studies have also shown that providing feedback regarding trunk angle and lower limb posture can also help to reduce the risk of LBP [13]. However, even though feedback about the trunk angle was completed in real time, lower limb posture feedback was performed by a personal trainer giving verbal instructions such as “use legs instead of back”. Thus, a system that could monitor the posture of the lower limbs without the need for a personal trainer was considered here. Particularly, we focused on foot position as an effective parameter for posture adjustment that can be easily adjusted [8,9]. However, despite the benefits of being able to monitor the position of the feet during manual lifting, no such monitoring system has been developed in the past.

Therefore, this study contributes to assessing foot position measurement using wearable sensors for the prevention of lower back pain during assistive motion for sit-to-stand. The highlight of this study is that a combination of few wearable sensors and machine learning can estimate foot position during manual lifting. The proposed foot position estimation method can be easily realized because it can be implemented through the use of only a single inertial sensor and shoe-type force sensors. Therefore, the proposed method could be applied for suitable assistive motion training to prevent LBP in nursing schools.

## 2. Materials and Methods

### 2.1. Proposed Method for Foot Position Measurement

#### 2.1.1. Architecture

**Figure 1:**
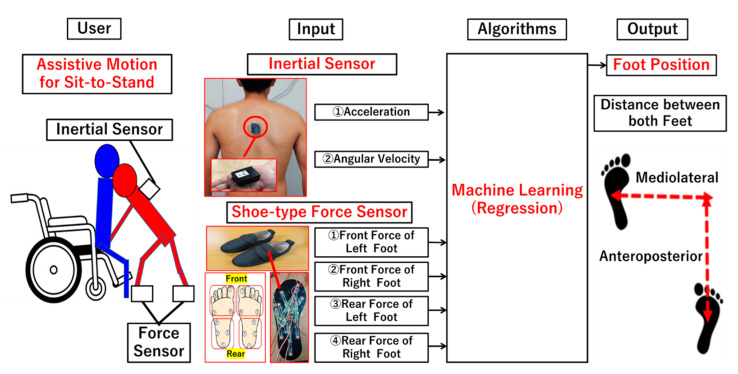
Architecture of the proposed method.

The architecture of the proposed method is shown in Figure 1. Wearable sensors are mounted on the trunk and both feet. These wearable sensors were selected based on aforementioned potentials and hypotheses in previous section. The inertial sensor on the trunk measures 3-axes accelerations and 3-axes angular velocities during assistive motion for sit-to-stand, whereas the shoe-type force sensors measure the front and rear force on both feet during manual handling. These sensor data are used for machine learning-based regression algorithms to estimate quantitative foot position. Anteroposterior and mediolateral distances between both feet are outputted quantitatively as the foot position. Components of this architecture are described in Figure 1.

#### 2.1.2. Wearable Sensors

The inertial sensor (LP-WS1104, Logical Product Co., Fukuoka, Japan) attached on the trunk measures the three-axis acceleration and angular velocity of the trunk and is used for the machine learning algorithm. The specifications of the inertial sensor were as follows: full range of sensor output in acceleration, ±5 G; acceleration sensitivity, 191.4 mV/g; full range of sensor output in gyro, 1500 degrees per second (dps); and gyro sensitivity, 0.8 mv/dps. 

The shoe-type force sensors consisted of eight force sensors on each insole. Four sensors were located on the forefoot, and the other four sensors were located from the midfoot to the hindfoot, as shown in Figure 1. The sum of the output values for each of the four sensors were calculated as the front and rear force on each foot (four total values). FlexiForce sensors (Tekscan Inc., South Boston, MA, USA) were selected for the proposed method given their thinness and flexibility [30,31]. The specifications of the force sensor were as follows: full range of sensor output, 445 N for one sensor; linearity, <±3% of full range of sensor output; and hysteresis, <4.5% of full range of sensor output. The noise of the force data was reduced via an amplifier circuit (FlexiForce Adapter 1120, Phidgets Inc. Calgary, AB, Canada).

The inertial sensor and data logger for the force sensors were synchronized using 2.4-GHz wireless communication in an IEEE802.15.4-based protocol and antenna (Logical Product Co., Fukuoka, Japan). The shoe-type force sensors measured front and rear vertical forces on each foot during manual handling. The sampling rates for these wearable sensors were set at 100 Hz. The inertial data were saved in a flash memory drive installed in the inertial sensor. Force data were saved in the flash memory drive installed in the data logger (Logical Product Co., Japan). These saved data were exported to a personal laptop personal computer via a USB cable.

#### 2.1.3. Machine Learning-Based Regression Algorithm

A machine learning regression model estimated the quantitative foot position from the wearable sensor data. For this purpose, several parameters were calculated from the time series data of each sensor signal to be used as input for the machine learning model. The extracted parameters included the mean, standard deviation, skewness, kurtosis, maximum, minimum, and root mean square of the data in each measurement. These parameters were determined based on previous studies using machine learning algorithm and wearable sensors [19,24,25]. These seven parameters were calculated for 3-axes accelerations, 3-axes gyro, and four force values (front and rear of each foot); thus, a total of 70 features were calculated for each trial. The proposed method estimates foot position based on an entire motion; thus, these parameters were extracted from an entire motion. 

Optimal machine learning algorithms depend on target movement and sensors. Thus, this study compared the outcomes of artificial neural network (ANN), Gaussian process, k-nearest neighbor (kNN), linear regression, and support vector regression (SVR) for the proposed method. These five machine learning algorithms were selected given their frequent use for wearable systems in previous studies [32,33,34,35,36]. In addition, ANN, Gaussian process, kNN, and SVR were expected to build regression models for nonlinear and complex data distribution [37,38,39,40]. These findings are more informative than the fact that these algorithms were simply tested. Furthermore, linear regression was expected to build a simple model for implementation advantages. The details of these machine learning algorithms are descripted in a later section.

#### 2.1.4. Foot Position

Our previous study found that both the anteroposterior and mediolateral distances are important for foot position adjustment aimed at reducing LBP during assistive motion for sit-to-stand [8]. Therefore, the proposed method outputs the anteroposterior and mediolateral distances between both left and right feet. Actual foot positions for the training and validation of the machine learning algorithm were measured using an optical motion capture system (OptiTrack, Corvallis, OR, USA) and optical makers on the both left and right heels. This study defined heel position as actual foot position without considering foot rotation and the center of the foot because our previous study found that changing heel position could reduce lumbar load during assistive motion for sit-to-stand [8]. The data from the optical motion capture system were sampled at 100 Hz to match the sampling rate of the wearable sensors. In addition, the optical motion capture system measured foot positions by means of a marker on each heel in the global frame coordinate.

#### 2.1.5. Expected Intervention

The proposed method will be applied for assistive motion training in each nursing school. The collection of the data to be used for machine learning can be collected from nursing students because nursing students repeat assistive motion during their lessons and during self-training [41]. Furthermore, previous studies have mentioned that improving assistive motion training for nursing students might be best approach for the long-term prevention for LBP because it is difficult to improve the assistive motion of experienced nurses [13]. The proposed method will be used by nursing students during assistive motion training. While undergoing assistive motion training, immediate feedback regarding foot position will be given immediately after one motion. If the foot position is unsuitable, the nursing students will be able to improve their foot position starting from the next motion. Nursing students will be able to acquire a suitable foot position via training with these processes.

### 2.2. Experiment for Evaluation of the Proposed Method

This study evaluated whether the proposed method could accurately measure quantitative foot position during actual manual handling. Moreover, we compared five common machine learning algorithms (ANN, Gaussian process, kNN, linear regression, and SVR) to determine the optimal algorithm for the proposed method. Details regarding this experiment are described below.

#### 2.2.1. Participants

Ten young male students (age 23.2 ± 1.03 years, height 171 ± 6.35 cm, weight 59.8 ± 5.14 kg, mean ± standard deviation) at the Kyushu Institute of Technology were recruited. The experiment was explained to all of the participants before participation, after which all participants signed an informed consent form before experimentation began. This study considered the idea that the proposed system could be implemented in nursing schools. In this intervention, training data would be collected at the same nursing school because different conditions such as the sizes or heights of beds and wheelchairs. Therefore, the training data size could be limited because of the number of students in each school. Thus, the use of only ten participants was allowed because this study did not expect to collect a large training dataset for generalization. Note that the participants had no experience as caregivers. All of the experimental procedures were conducted in accordance with the Declaration of Helsinki and the Ethics Committee for Human Research of the Graduate School of Life Science and Systems Engineering, Kyushu Institute of Technology (approval number: 19-05).

#### 2.2.2. Experimental Procedure

**Figure 2:**
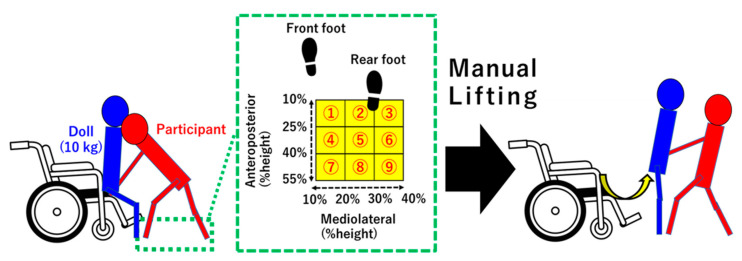
Assistive motion for sit-to-stand with different foot positions.

After putting on the wearable sensors (inertial sensor and shoe-type force sensors) for the proposed method, the participants were asked to lift the lower back of a doll (height 145 cm, weight 10.0 kg) from a wheelchair (height 45.0 cm). This assistive motion for sit-to-stand is shown in Figure 2. This motion, which entails the patient being picked up from the lower back, is common assistive motion [41]. The participants executed a sit-to-stand movement with the doll (simulated patient). The doll was lighter than an actual human due to concerns about the patients placing substantial stress on their lumbar area. After practicing this manual lifting motion (10 min before measurement), measurements were obtained while the participants performed manual lifting using nine different foot position patterns, which are presented in Figure 2 and Table 1. Foot distances were normalized to the body height of each participant (unit: %height) in order to avoid the effect of the different body heights between the participants. The left foot was fixed to the anterior, whereas the right foot moved to adjust the foot distances for each foot position pattern. Previous studies indicate that caregivers should stand close to the patient to reduce the lumbar load [42]. Thus, the left foot was always fixed to the same spot between the footrests of the wheelchair during each trial. As shown in Table 1, these foot patterns included different combinations of anteroposterior (10–25, 26–40, and 41–55 %height) and mediolateral (10–20, 21–30, and 31–40 %height) foot distances. These foot positions were defined for collecting various foot position data. The participants repeated the manual lifting technique for over five trials for each foot position (a total of 45 trials for each participant), changing the foot distances for each trial in range of each foot position pattern by themselves. Wearable sensors and optical motion capture systems (eight cameras, OptiTrack, Corvallis, OR, USA) measured the assistive motion for sit-to-stand with a 100-Hz sampling rate. The optical markers for the motion capture system were mounted on the heels of both shoe-type force sensors.

#### 2.2.3. Data Analysis

The proposed method was evaluated after obtaining all of the measurement data from 450 trials. The input parameters for the machine learning model (mean, standard deviation, skewness, kurtosis, maximum, minimum, and root mean square) were calculated for all wearable sensor signals (front and rear force of each foot, 3-axes acceleration, and 3-axes angular velocity) using MATLAB R2020b (MathWorks, Natick, MA, USA). The actual foot position values (anteroposterior and mediolateral feet distances) were calculated using optical motion capture data and the 3D analysis software VENUS 3D R (Nobby Tech. Ltd., Tokyo, Japan). Foot position data were normalized to the body height of each participant.

Five machine learning algorithms (ANN, Gaussian process, kNN, linear regression, and SVR) were implemented and were verified using the data mining software WEKA 3.6 (University of Waikato, Hamilton, New Zealand) [43]. The specifications and parameters of each machine learning algorithm are shown in Table 2, Table 3, Table 4, Table 5 and Table 6. The radial basis function (RBF) kernel was applied for Gaussian processes and SVR given that this kernel was used in previous studies related to wearable sensing [44,45].

The accuracy of the proposed method was evaluated from 450 trial data obtained from 10 participants. The accuracies of each machine learning algorithm for the proposed method were calculated using 10-fold cross validation. In the 10-fold cross validation, the dataset was randomly divided into 10 subgroups. Nine subgroups were used as training data, and one subgroup was used as test data. This process was repeated 10 times, and each subsample was used in turn as the test data. As mentioned previously, this study did not expect to prepare a big training dataset for generalization because we considered the fact that the proposed method would be used in different nursing schools. Because of this, it is important that the training data be collected in school that the method is being used in because each school has different conditions. Thus, we considered that these data size and validation processes were suitable. All of the estimated foot positions were obtained from the 10 calculations based on the 10-fold cross validation. The root mean square error (RMSE) between the estimated and actual foot positions was calculated as the error of the proposed method, selecting the machine learning algorithm with the smallest RMSE value as the optimal algorithm. Furthermore, the RMSE values were compared for three feature patterns (only inertial sensor, only shoe-type force sensors, and all wearable sensors) to select the optimal wearable sensors. Through such processes, the optimal combination of wearable sensors and machine learning algorithms can be determined.

Statistical analyses were performed to determine the optimal combination of wearable sensors and machine learning algorithms using EZR 1.32 (Division of Hematology, Saitama Medical Center, Jichi Medical University, Saitama, Japan) [46]. Significant differences between the error of the proposed method and zero were evaluated using the paired t-test (significance level: *p* < 0.05). In addition, power analyses were performed for the paired t-test (α = 0.05). Pearson’s correlation between the proposed method and actual values was calculated as the accuracy of the proposed method (significance level: *p* < 0.05).

The Bland–Altman plot, which represents the mean (horizontal axis) and difference (vertical axis) between estimated and actual values, was used to evaluate fixed and proportional errors of the proposed method using the optimal algorithm [47]. The limitation of agreement (LOA) of the vertical axis in the Bland–Altman plot was used to evaluate the fixed errors. Accordingly, an LOA that does not include zero indicates a fixed error [47]. Pearson’s correlation between the mean (horizontal axis) and difference (vertical axis) of the Bland–Altman plot was calculated to evaluate the proportional error (significance level: *p* < 0.05). Significant correlations between the mean and difference indicates a proportional error [48].

## 3. Results

### 3.1. Data Specification

**Figure 3:**
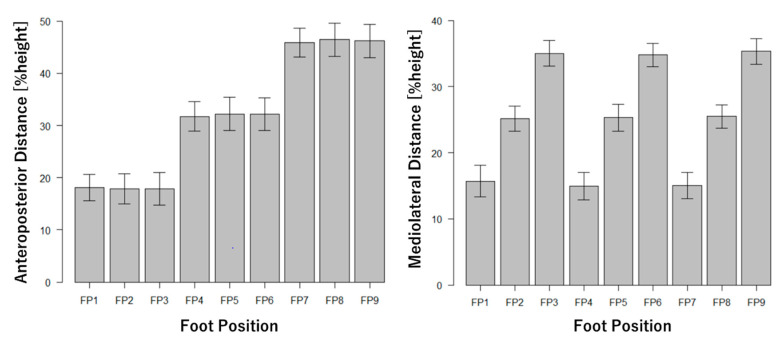
Foot distances of the nine foot positions obtained from the optical motion capture system. %height is feet distance normalized to the body height of each participant. Values are mean ± standard deviations. FP: foot position.

**Figure 4:**
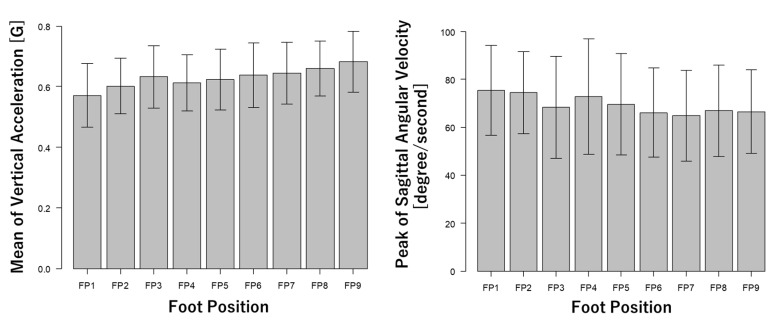
Mean of vertical acceleration and peak of sagittal angular velocity obtained from inertial sensor. Positive of acceleration mean superior direction. Positive value of angular velocity mean trunk extension. Values are mean ± standard deviations. FP: foot position.

**Figure 5:**
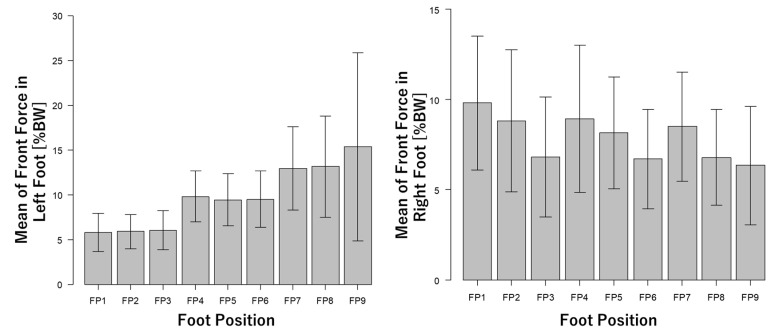
Mean of front force in left and right foot obtained from shoe-type force sensor. %BW is force normalized to the body weight of each participant. Values are mean ± standard deviations. FP: foot position.

The anteroposterior and mediolateral foot distances measured by the optical motion capture system are shown in Figure 3. Measured foot distances (Figure 3) were satisfied for the nine different foot positions defined in Table 1 and Figure 2. Figure 4 shows vertical acceleration and sagittal angular velocity obtained from the inertial sensor. Figure 5 shows the front force of the foot obtained from the shoe-type force sensor. The results of the inertial data show that vertical accelerations were increased by a longer foot distance (Figure 4). On the other hand, the sagittal angular velocities were decreased by a shorter foot distance (Figure 4). The results of the force data show that the forces of the left (anterior) foot were increased by a longer foot distance. On the other hand, the forces of the right (posterior) foot were decreased by a shorter foot distance. These results indicate that inertial and force data were affected by the foot position changing.

### 3.2. RMSE Values

The RMSE values of the anteroposterior and mediolateral foot distances between the proposed method and actual foot position are shown in Table 7 and Table 8. Accordingly, the RMSE values of the proposed method using all wearable sensors (inertial sensor and shoe-type wearable sensors) were the smallest for both anteroposterior and mediolateral foot distances in three feature patterns. Moreover, the RMSE values of the proposed method using Gaussian process were the smallest for both anteroposterior and mediolateral foot distances in the five algorithms. Based on the aforementioned results, the combination of all of the wearable sensors and the Gaussian processes was determined to be the optimal combination of features and algorithms for the proposed method. The proposed method using the optimal combination of features and algorithm could measure the anteroposterior and mediolateral foot distances with less than 6.5 %height RMSE (anteroposterior: 6.06 %height; mediolateral: 6.30 %height).

### 3.3. Statistical Results

The results of the statistical analyses are summarized in Table 9. The errors of the proposed method (Mean ± S.D.) are shown in Table 9. Accordingly, no significant differences in both the anteroposterior and mediolateral foot distances were observed between the error of the proposed method and zero (*p* > 0.05). Moreover, significant correlations between the proposed method and actual values were observed for both the anteroposterior and mediolateral foot distances (*p* < 0.05).

### 3.4. Bland–Altman Plot

**Figure 6:**
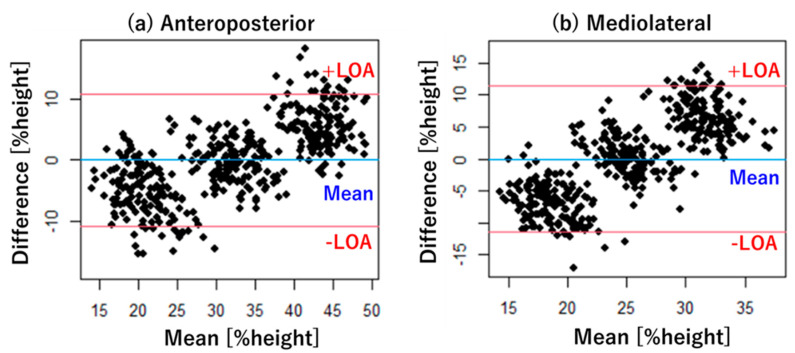
Bland–Altman plots between the proposed method and actual values. The proposed method used all wearable sensors and Gaussian process. (a) Anteroposterior foot distance; (b) mediolateral foot distance. Blue line: mean of difference; red line: limitation of agreement (LOA). %height is feet distance normalized to body height of each participant.

Bland–Altman plots, which are presented in Figure 6, were used to evaluate the proposed method using all of the wearable sensors and Gaussian process. The statistical results for the Bland–Altman plots are detailed in Table 10. The LOA of the Bland–Altman plot included zero for both the anteroposterior and mediolateral foot distances, suggesting the absence of fixed errors in the proposed method. However, significant correlations were noted between the difference and mean of the Bland–Altman plots for both the anteroposterior and mediolateral foot distances (anteroposterior: r = 0.682 and *p* < 0.05; mediolateral: r = 0.758 and *p* < 0.05), suggesting proportional errors in the proposed method.

## 4. Discussion

### 4.1. Validity of the Proposed Method

Our results showed that the proposed method using the optimal combination of wearable sensors and machine learning algorithm could accurately measure anteroposterior foot distances with an RMSE of 6.06 %height (Table 7). One previous study showed that the number of steps can be a useful measurement unit to provide feedback regarding foot position during manual handling [20]. In addition, the number of steps has enough resolution because our previous study showed that lumbar load might be affected by changing the foot position with at least 10 to 15 %height [8]. Thus, since the anteroposterior RMSE value is shorter than the foot length (foot or shoe size) [49,50], the proposed method could be applied to provide feedback about the anteroposterior foot position using the number of steps (foot or shoe). For example, when the measured anteroposterior foot distance is shorter by approximately 10 %height than the optimal distance, the system can provide feedback that says, "please back your rearfoot up by one step”. On the other hand, the RMSE of the proposed method for the mediolateral foot distance was 6.30 %height (Table 8), which was longer than the foot width [50]. Thus, future studies should attempt to minimize measurement errors in the mediolateral foot distances.

Our statistical analyses showed a significant correlation between the proposed method and the actual foot position in both the anteroposterior and the mediolateral feet distances (Table 9). In addition, there were no significant differences that were seen for the errors of the proposed method and zero (Table 9). These results indicate that the proposed method involving the use of wearable sensors and a machine learning technique can effectively measure foot positions. Moreover, a significant correlation between the proposed method and the actual foot position in the mediolateral foot distance suggests the possibility of improving errors in the mediolateral foot distance of the proposed method.

The LOA of the Bland–Altman plots showed no fixed error in both the anteroposterior and mediolateral foot distances (Figure 6 and Table 10), suggesting that the proposed method could be used to measure foot position during manual lifting without bias or systematic errors. Nonetheless, correlations between the Bland–Altman plots showed proportional errors in both the anteroposterior and mediolateral foot distances (Figure 6 and Table 10), indicating the need to focus on proportional errors for the future improvement of the proposed method.

The aforementioned results therefore suggest that the proposed method can be utilized for the monitoring of and giving instructions on quantitative foot positions during assistive motion for sit-to-stand to prevent LBP among caregivers. Furthermore, these results highlight the direction of future improvements in the proposed method.

### 4.2. Wearable Sensors

The RMSE values showed that both the inertial sensor and the shoe-type force sensor were useful for the proposed method (Table 7 and Table 8). In addition, inertial and force data were affected by the changing foot position (Figure 4 and Figure 5). The inertial sensor on the trunk could perceive differences in the foot position given that trunk movement is affected by foot position during manual handling [19,51]. Moreover, the shoe-type force sensors could measure the force changes caused by the foot position given that the force distribution on the insole changes based on the foot position during manual handling [25]. Therefore, both an inertial sensor and the shoe-type force sensors were considered for the proposed method. Additionally, these wearable sensors can be combined to measure lumbar load and arm movement during manual handling [19,26], thereby making them useful for various measurements aimed at preventing LBP for occupational health.

Nonetheless, using only both inertial sensor and shoe-type sensors for the proposed method allowed us to be able to measure the anteroposterior and mediolateral foot distances with less than 7.5 %height RMSE by means of Gaussian process (Table 7 and Table 8). Improving on such errors by modifying features or algorithms could perhaps allow the use of only either an inertial sensor or shoe-type force sensors for the proposed method. Indeed, reducing the number of sensors would increase user comfort. Furthermore, using only a single inertial sensor for the proposed method can be viable through the use of smartphones given the reliable and valid inertial sensors in current smartphones [52,53]. According to the current fixation condition for the inertial sensor, smartphones would need to be attached to the users back with a belt. If smartphones are able to be placed in a chest pocket, then the method would be more user friendly. However, the accuracy of the proposed method when placing a smartphone in a chest pocket should be investigated. Such combinations and applications for wearable sensors would ultimately be based on the usability and accuracy required by each user.

### 4.3. Machine Learning Algorithms

The proposed method using Gaussian process was able to measure both the anteroposterior and mediolateral foot distances with the smallest RMSE (Table 7 and Table 8). The reason for these results is that Gaussian process are suitable for machine learning with a small dataset, such as that used in this study. This is because Gaussian process can change the margin of fitting based on the number of data points [37]. Machine learning algorithms that allow the use of small datasets can be useful for actual workspaces given the ease of data acquisition. Therefore, Gaussian process can be considered the optimal machine learning algorithm for the proposed method. On the other hand, the RMSE values of several algorithms were similar. Therefore, there is a possibility that several algorithms other than Gaussian process could also be applied for the proposed method. Future work should continue to explore the optimal algorithm by considering other factors such as implementation or calculation speed.

### 4.4. Limitation and Future Works

The proposed method was evaluated by performing assistive motion for sit-to-stand in a laboratory setup. Future studies should evaluate the proposed method in various motions in actual nursing students. An additional limitation is that the foot positions were measured using the heel marker of a global coordinate frame. A single marker on the heel does not approximate a centroid on the foot, and the foot rotation might change the center position of the foot. Moreover, another limitation of this experiment was that the left foot was fixed between the footrests of a wheelchair. There is the possibility that an actual caregiver cannot fix their footing during an assistive motion. The accuracy of the proposed method might be affected by this unstable foot position. Thus, the proposed method should be tested for unstable foot positions during assistive motion. In this study, was another limitation was that the 10 kg doll used in this experiment is lighter than actual patients. This creates a problem, as the method is less reliable due to the difference between the doll used in the experiment and actual patients. According to previous studies, the COP velocity is affected by changing weight [54]. Thus, patient weight might affect the force data obtained from the shoe-type force sensors. Thus, the effect of patient weight on the proposed method should be investigated in future work. In the experiment, each data included the start and end points. The start and end points of assistive motion should be extracted automatically in order for the system to be useful. Previous studies developed an automatic recognition method for patient handling tasks including assistive motion for sit-to-stand using footwear sensors, which were similar to our shoe-type force sensors [55,56]. In future work, these previous methods might be combined with our proposed method for automatic foot position estimation.

As mentioned previously, errors regarding the mediolateral foot width should be improved. Future studies must examine feature selection methods [57,58] and other machine learning algorithms [59,60] to improve the accuracy of the proposed method. Moreover, linear-based solutions to reduce the proportional error of the proposed method must be developed. The regression model of this study could only be applied for a specific learned task. In future work, a new generalized regression model that learns various patient handling tasks should be considered. Furthermore, readable models such as a biomechanical link model [61,62] should be combined to improve accuracy. The proposed method estimates foot position from an entire motion. In this case, our future system will give feedback regarding foot position immediately after assistive motion. When the system gives feedback indicating that the foot position is not suitable, the caregiver will be able to modify their foot position for the next motion. However, it is desirable that measurements and feedback be made prior to completing an assistive motion. Thus, future work should focus on foot position measurement in the initial posture. 

After improvements and further evaluation, monitoring and instruction systems for foot position during manual lifting must be implemented. However, problems remain for system integration using a wireless sensor network. This study could not consider real-time processing, transmission time, and network lifetime. In future work, the proposed method should be implemented for real-time processing based on recent frameworks [63,64]. For example, Coviello et al. proposed a framework for wireless synchronization based on sending and transmission times [63]. Moreover, Sakuru et al. suggested that sink node selection algorithms based on network lifetime could be applied for wireless sensor networks [64]. Our proposed system will be implemented using a wireless sensor network with these frameworks [63,64]. Furthermore, a feedback method for foot position using the proposed method should be developed. Foot position might be corrected by means of audio or using a haptic device as well as previous feedback methods used to correct the trunk angle [10,11,12,13].

Our previous study found that foot position adjustment could reduce lumbar loads during assistive motion for sit-to-stand by using lower limb muscles [8]. In this previous study, the participants performed lifting with the legs and not the back without other instructions [8]. Thus, we considered that a feedback system for foot position would be useful to realize assistive motion using the lower limbs. However, there is a possibility that the best instruction to generate optimal assistive motion to prevent lower back pain requires feedback for another postural parameter, such as the trunk angle. Thus, future studies should consider a combination of both foot position and trunk angle for more effective intervention.

## 5. Conclusions

This study proposed and evaluated a new method for measuring foot position during assistive motion for sit-to-stand in order to prevent LBP among caregivers. The proposed method can be utilized for occupational health for caregivers given that it only uses a few wearable sensors. The following are the contributions of the current study:

- The proposed method can measure quantitative foot positions using a single inertial sensor, shoe-type force sensors, and a machine learning algorithm.
- The proposed method was evaluated using RMSE values, statistical analysis, and Bland–Altman plots.
- Optimal combinations of wearable sensors and machine learning algorithm were explored for the proposed method.
- The experimental results showed that a combination of both inertial and shoe-type force sensors and Gaussian process is the optimal combination for the proposed method.
- The RMSE values and statistical results indicated that the proposed method could measure foot position during assistive motion for sit-to-stand.
- Bland–Altman plots showed that the proportional error should be improved in the proposed method.

The proposed method will certainly contribute to wearable monitoring and instruction systems for manual lifting to prevent LBP among caregivers. We believe that the proposed method can be applied in nursing schools that require training for assistive motion for sit-to-stand.

## Figures and Tables

**Table 1:** Foot distances of the nine foot positions during manual lifting.

		Mediolateral Distance [%Height]		
		10–20	21–30	31–40
**Anteroposterior Distance [%height]**	**10–25**	Position 1	Position 2	Position 3
	**26–40**	Position 4	Position 5	Position 6
	**41–55**	Position 7	Position 8	Position 9

%height is feet distance normalized to body height of each participant. Nine foot positions have different combination of anteroposterior and mediolateral distance. Each anteroposterior and mediolateral distance has range of value.

**Table 2:** Specifications and parameters of artificial neural network (ANN).

Specification/Parameter		Status/Value
Number of Layer	Input Layer	1
	Hidden Layer	1
	Output Layer	1
Number of Neuron / Node	Input Layer	At most 70 (based on features)
	Hidden Layer	At most 70 (based on features)
	Output Layer	1
Activation	Hidden Layer	Sigmoid
	Output Layer	Linear
Learning Rate		0.3
Momentum		0.2
Training Method		Back Propagation

**Table 3:** Specifications and parameters of Gaussian process.

Specification/Parameter	Status/Value
Kernel	RBF Kernel
Hyperparameter γ	1.0

**Table 4:** Specifications and parameters of k-nearest neighbor (kNN).

Specification/Parameter	Status/Value
Weight	Uniform
Distance	Euclidean Distance
Hyperparameter K	1

**Table 5:** Specifications and parameters of linear regression.

Specification/Parameter	Status/Value
Feature Selection	M5 Method
Ridge Parameter R	1.0 × e^−8^

**Table 6:** Specifications and parameters of support vector regression (SVR).

Specification/Parameter	Status/Value
Training	Sequential Minimal Optimization
Kernel	RBF Kernel
Hyperparameter γ	0.01

**Table 7:** Root mean square error values of anteroposterior foot distances.

Feature Pattern	Algorithm	RMSE [%Height]
Only Inertial Sensor	ANN	11.5
	Gaussian process	**7.20**
	kNN	8.43
	Linear regression	8.40
	SVR	9.69
Only Shoe-type Force Sensors	ANN	10.6
	Gaussian process	**7.10**
	kNN	9.14
	Linear regression	7.85
	SVR	8.02
All Wearable Sensors	ANN	7.93
	Gaussian process	**6.06**
	kNN	6.89
	Linear regression	6.62
	SVR	7.30

%height is feet distance normalized by body height of each participant. RMSE: root mean square error; ANN: artificial neural network; kNN: k-nearest neighbor; SVR: support vector regression.

**Table 8:** Root mean square error values of mediolateral foot distances.

Feature Pattern	Algorithm	RMSE [%Height]
Only Inertial Sensor	ANN	11.0
	Gaussian process	**7.16**
	kNN	8.72
	Linear regression	7.72
	SVR	8.03
Only Shoe-type Force Sensors	ANN	10.9
	Gaussian process	**6.50**
	kNN	8.80
	Linear regression	6.88
	SVR	7.39
All Wearable Sensors	ANN	9.77
	Gaussian process	**6.30**
	kNN	8.01
	Linear regression	6.80
	SVR	7.00

%height is feet distance normalized by body height of each participant. RMSE: root mean square error; ANN: artificial neural network; kNN: k-nearest neighbor; SVR: support vector regression.

**Table 9:** Statistical results.

Foot Position		Paired *t*-Test (Error vs. Zero)	Correlation (Estimated vs. Actual)
	Significant Difference	Power	
Anteroposterior	N.S.	0.0284	0.891 *
Mediolateral	N.S.	0.0289	0.692 *

%height is feet distance normalized to the body height of each participant. S.D.: standard deviations. Significant differences were evaluated using paired *t*-tests. Correlation was calculated using Pearson’s correlation. N.S.: not significant (*p* > 0.05); *: significant (*p* < 0.05).

**Table 10:** Statistical results for the Bland–Altman plots.

Foot Position	Limitation of Agreement [%Height]	Correlation (as Proportional Error)
Anteroposterior	−10.9 to 10.9	0.682 *
Mediolateral	−11.4 to 11.3	0.758 *

%height is feet distance normalized to the body height of each participant. Correlation was determined using Pearson’s correlation. Correlation between Bland–Altman plots was calculated as the proportional error. *: significant (*p* < 0.05).

## Data Availability

Data are stored in a password-protected PC in the Kyushu Institute of Technology.
